# The benefits of nursing professional governance nursing research and evidenced-based practice councils for new graduate nurses

**DOI:** 10.1016/j.dialog.2024.100192

**Published:** 2024-09-02

**Authors:** Cheryl Green, John Brennan, Lauren Koscal, Emma Sears, Jessica Munoz, Evelyn Jacovino, Lauren Thayer, Todd Allen Lane, Elizabeth Dos Santos

**Affiliations:** Bridgeport Hospital- Yale New Haven Health, 267 Grant Street, Bridgeport, Connecticut 06610, United States

**Keywords:** Nursing professional governance council, New graduate nurses, Nurse leader, Nursing research and evidenced-based practice councils

## Abstract

New graduate nurses are an untapped population for nursing professional governance and nursing research and evidenced-based practice councils. New graduate nurses can offer new insights into the management of patient care and nursing workflow. By educating new graduate nurses about the benefits of nursing research and evidence-based practice councils at the start of their careers, an early standard of excellence in the empowerment of applying evidenced-based practice principles to improve patient care quality can occur. This narrative literature inquiry explores new graduate nurses perspective of research and evidence-based practice councils, with the targeted goal of engagement for professional and personal growth as a nurse leader.

## 1 Introduction

New graduate nurses entering acute care hospital settings can provide fresh insights and new perspectives on nursing workflow and management of patient care as they integrate into pre-existing health care teams on clinical units. Having recently left academic studies in nursing science and supervised clinical experiences with their didactic and clinical faculty, new graduate nurses, upon completion of orientation to their clinical unit(s) and the organization, are great candidates for early engagement in the participation within nursing professional governance and nursing research and evidenced-based practice councils [18], [19].

## 2 Background

### 2.1 Current subsets for nurses

There are three subsets of hospital employed nurses that are potential candidates for participation within nursing research and evidenced-based practice councils. These include, care providers who are unaware of the existence of research and evidenced-based practice councils', nurses seeking career advancement, and nurses who recently completed their nurse residency program [1]. A logical place to start would be to actively recruit the unaware nurse. Advertising via screen shots, emails to nurses or postings on clinical units' bulletin boards help entice nurses interested in the application of nursing research and unit-based quality improvement projects [4], [5]. Some nurses are unaware of extra activities that are beyond their normal working environments. As a result, unengaged, but highly motivated nurses are an underutilized resource waiting to be capitalized on.

Often, nursing research is lacking in the application of current standard nursing skills and practice. As a result, a research council may not be in the forefront of bedside nurse leaders' thoughts or perspectives in their daily care of patients, interactions with families, healthcare providers and other disciplines. However, exemplifying the importance of nursing research with opportunities to improve patient care and nursing delivery of quality health care, can help with the enrollment of new nurses to a council [26].

Another pool of available candidates for nursing professional governance nursing research and evidence-based councils lies in nurses returning to school for advanced degrees. There has been an increase in the number of nurses going back to school for programs such as certifications, master's in nursing, a doctorate in nursing, and other career advancements. Often, there are clinical and practical portions of these programs that are required to graduate. Nursing professional governance provides councils for these like-minded, motivated, individuals to meet and discuss research and quality improvement practices. Moreover, research and evidence-based practice councils provide opportunities to engage and fulfill organizational and institutional requirements [10].

## 3 Purpose

This is an exploration by a nursing research and evidenced-based council located in a New England acute care hospital, of how nursing professional governance with nursing research and evidenced-based practice councils can personally and professionally benefit new graduate nurses entering acute care hospital systems. The purpose of this exploration is to aid new graduate nurses in their transition to practice as registered nurses, while preparing them to engage in evidenced-based practice and research at the bedside with patients to improve patient outcomes, and with their nursing peers to improve workflow. The latter may also help new graduate nurses build confidence and feel supported as they embark on their new career [18], [19].

### 3.1 Transition to practice

Gautam et al. [14] applied a qualitative descriptive method with the use of semi- structure interviews with 10 participants in a study of new graduate nurses' experiences with transition to practice. Gautam et al. [14] noted that new graduate nurses found the transition to professional practice to be a stressful experience whereby they felt unsupported, lacked confidence when confronted by the realities and complexities of being a registered nurse and providing care to patients. The new graduate nurses reported having to gather their own strength to survive the newness of their nursing practice experience.

As new graduate nurses complete their undergraduate education; they leave academia to embark on their career as licensed registered nurses. Having to apply skills once demonstrated in the safe confinement of a nursing laboratory with oversight for safety and competency verification by nurse educators in practice settings, new nurses can feel anxious and fearful of doing harm to patients in their care.

Transition programs like the Vizient and the American Association of Colleges of Nursing (AACN) nurse residency programs, provide new graduate nurses the opportunity to receive support from their peers within the context of a cohort and larger community [41]. Additionally, mentoring by nurse leaders [29] acting as resident facilitators within nurse residency programs, can help new graduate nurses develop a sense of belonging and connection to the healthcare organization. With a connection to the organization, new graduate nurses are more likely to have a higher rate of retention [2, 29].

Vizient and AACN nurse residency programs also require evidenced-based practice projects to be identified, researched, and implemented during new graduate nurses' participation in the nurse residency program [2]. The evidenced-based practice projects are identified and implemented on clinical units that the new graduate nurses are receiving orientation on. This experience with research of projects that are evidence-based practice can prepare new graduate nurses for participation on nursing research and evidence-based practice councils.

Orton [31] emphasizes the importance of shared governance in supporting nursing autonomy, which is particularly beneficial for new graduate nurses. Shared governance models empower nurses by involving them in decision-making processes, and enhancing their professional development and sense of control over their practice [1], [5], [7]. This empowerment can alleviate feelings of helplessness and improve job satisfaction, making the transition to practice smoother for new graduates [31]. Therefore, a comprehensive approach that includes residency programs, mentoring, preceptorship, and shared governance is essential to successfully transitioning new graduate nurses into clinical practice.

Before exploring the scope of a nursing research and evidenced-based practice council and its application to new graduate nurses, it is imperative to have an understanding of what a nursing practice council represents. Shared governance has been a staple of institutional nursing for over 40 years. Though design and structure may vary depending upon the organization, the overall mission of these organizing bodies is relatively similar. These groups serve to bring nurses' voices and concerns to the forefront of executive attention (Kremer et al., [7]). Historically, this aspect of empowering the employee voice (EV) has been a cornerstone of shared governance. This idea expands to the actions and behaviors critical to addressing council goals and objectives. EV, in this case, is based on the individual's morals, experience, and perspectives. EV allows for a level of holistic processing to address varying aspects of shared issues, ultimately leading to structural change and improvement [22], [24], [25]. Structural change and improvements can happen at an organizational level or be delegated to committee work [23]. Often, some subgroups take different focuses based on clinical and administrative expertise. Additionally, it is common for there to be an interdisciplinary composition to better address mutual issues. Regardless, these groups serve as a medium for nurses and other applicable disciplines to promote change and manage practice concerns.

This arrangement is multifaceted because nursing professional governance practice councils foster a symbiotic relationship between nurses and the organization. Nurses, for their part, field their concerns and are given a forum to utilize EV to address challenges amongst peers in a constructive atmosphere. Often, these issues are addressed with a multidisciplinary approach and allow clinicians to build connections amongst contemporaries. Furthermore, these staff exemplify themselves as leaders and are more likely to promote evidence-based practice (Kremer et al., [24]; [23]). From the organizational perspective, the institution is made aware of frontline issues that can be financially volatile. Having practice councils also has benefits in improving staff engagement and minimizing employee turnover. Allowing an assembly to voice concerns can be beneficial to high-stress occupations such as nursing. Finally, participating staff are often empowered to achieve and share best practices throughout the organization. There is often a focus on evidence-based improvement and this commitment to quality provides exponential benefits [30]. Exploring the relationship that practice governance provides can help illuminate the criticality of this assembly.

With this information in mind, interdisciplinary councils such as a nursing research council are often overlooked in the larger scheme of the nursing occupation. This perceived apathy can be attributed to the demands of the nursing profession and staff fatigue. Yet, these councils are critical now, more than ever. These groups promote best practices and a culture of continued learning. Moreover, having shared governance is a requirement for other institutional accolades such as Magnet designation.

The recent coronavirus 2019 (COVID-19) pandemic highlighted the importance of having a dynamic and versatile workforce. More specifically, local governments are looking at staffing ratios to ensure safe patient care. This legislation promotes frontline nurses' voices being heard [9,27]. A shared governance council can address these pertinent concerns. Nursing professional practice councils will play a more active role in the future of healthcare.

Nursing professional governance is a platform that allows new graduate nurses to collaborate in making policy and practice decisions that have a direct impact on the work environment. This shared governance model allows nurses to make evidence-based decisions to improve quality of care, patient outcomes, and positively impact both patient satisfaction and employee engagement [8], [15], [17]. This forum also provides nurses with the opportunity for professional growth while taking ownership of their own practice.

As more healthcare institutions seek magnet designation, it is imperative that research and evidenced-based practice projects be nurse-led. Empowering bedside leaders is necessary not only for the improvement in the health outcomes of patients provided care within a healthcare system or network, but also has the potential to have a national and international impact when results are disseminated [13]. Nursing science improves the lives of patients, families, and significant others and the workflow and productivity of nurses working within a variety of specialty areas.

Magnet designation through the American Nurses Credentialling Center (ANCC), sets an organizational precedence for registered nurses to enhance career development with furthering of their education, and expertise in their practice areas through specialty certifications. As a result, bedside nurse leaders become more innovative, and patients receive evidenced-based care by registered nurses [2].

## 4 Methods

This is a narrative literature review that was conducted May through June of 2024 on the topic of the recruitment of new graduate nurses to participate in nursing professional governance and the joining of the nursing research and evidence-based practice councils as a participating member. The readiness and preparation of new graduate nurses to join this particular council, was examined, as well as best practices to support new graduate nurses in transitioning to nursing practice and council service.

The CINAHL database was searched using the following key words: new graduate nurse, nursing professional governance, nursing research and evidence-based practice councils, and acute care hospitals. The CINAHL search yielded a total of 550 articles. The articles ranged from topics around empowering bedside leaders with council membership involvement to inclusion of new graduate nurses in research and evidenced-based practice quality improvement projects. The latter topic themes were cohesive and were simultaneously present in the articles. Interestingly, the majority of these articles were written in the early 2000s making most over 18 years old. Hence, while the articles provided valuable insight into new graduate nurses participation in research and evidence-based practice councils and unit-based projects, current approaches for engagement and recruitment of this population were sparse [3,[11], [12], [13],^16^,^18^,^19^,^36^,^40^].

## 5 Results

Before exploring engagement and recruitment opportunities targeting new graduate nurses for council membership, it is important to understand the application process of shared governance. For example, within the Bridgeport Hospital Network, a part of the Yale New Haven Health System, it is expected that individuals applying to shared governance have at least six months experience before volunteering to participate. In the context of organizational training, the first three months of a new hire are spent onboarding the new nursing staff member. Having an additional three-month waiting period can allow time to acclimate the new nurse hires to their clinical practice environments. Shared governance is usually a voluntary process that involves the individual participating in a council that corresponds with their work (e.g., Medical-Surgical Council) experience [21]. Often, these opportunities are used to advance up the career ladder as well as engage motivated individuals for personal and professional growth. With this in mind, how does an abstract discipline such as a nursing research council attract new members?

### 5.1 New graduate nurses

New graduate nurses represent a subpopulation of nurses that would benefit from participating in an evidence-based research council. Vizient/AACN residency programs last a year [41]. During this time nursing practices are still mailable enough to learn new skills and embrace differing perspectives. Within a part of new nurse residency, there is a quality improvement initiative associated with building resilience to burnout via participation within a cohort of like-minded new graduate nurses. Hence, the new graduate nurse learns that there is strength in team-building and team cooperation; for example, the completion of residency quality improvement projects with team members from their cohort [1], [3]. Because of the population characteristics of this group, they are the ideal candidates to recruit from. The nursing quality projects the new graduate nurses initiated during their residency programs can be the start of larger hospital improvement initiatives [28,34].

Through advertisement and sponsored activities to promote recruitment (e.g., council fairs), early engagement with new graduate nurses can be initiated. Council members can also request invitation via nurse managers, to share information and benefits of council membership during staff meetings. An explanation must be provided to new graduate nurses that although participation within a nursing research and evidence-based practice council is an additional occupational requirement, it can contribute to career advancement. Exploration of the literature that is pertinent to the nursing practice of council members helps bring relevancy of research and quality improvement projects to the bedside. The wider-reaching and more pertinent the topic, the better [4]. Roles and responsibilities for those nurses on the continuum of their educational journey are enhanced. By addressing scholarly topics from a holistic perspective council members will feel more engaged and empowered. [6].

Pertinent to the success of a nursing research and evidenced-based practice council is showcasing the success of new members [3], [13]. Often as a biproduct of new research, improved practices and designs are developed, as a result, councils need to recognize the success of its members. Not only does this highlight the work of coworkers, but provides an example of the value of dissemination of one's work and the importance of collaboration on projects [42]. This in turn, attracts more interested individuals and creates a sustainment state that is self-sufficient. The actions of a nursing research and evidence-based practice council are just as critical as the preferred candidates the group is attempting to attract. Having a keen insight into the activities critical to promotion within the healthcare organization can help attract new members.

## 6 Discussion

Research demonstrates that nursing professional governance promotes bedside leaders by empowering them to participate in decisions that may contribute to the profession of nursing, the healthcare institution, and the quality of services offered [20]. As a result, it is crucial for a nursing research and evidence-based practice council to incorporate the appropriate mix of academic and bedside leaders to ensure the council's success. Appropriate committee makeup helps to promote development of positive psychological capital, which is beneficial to innovation, professional advancement, and the development of research [32], [33], [35]. Hence, the new graduate nurse is an invaluable council member. Through the exploration of a transition to practice, application of a shared governance council, implications of research and quality improvement projects at the unit and systems level, and the recruitment of new graduate nurses, councils can develop learning objectives that can prepare council members to begin the work of clinical care enhancement [18]. Having a nursing research and evidence-based practice council can bring a plethora of benefits to healthcare organizations interested in investing in the improvement of patient outcomes, nurse retention, nurses professional and personal growth, and readiness for evaluation by local Departments of Public Health, The Joint Commission and Magnet designation [37], [38], [39], [40].

## CRediT authorship contribution statement

**Cheryl Green:** Writing – review & editing, Project administration, Conceptualization. **John Brennan:** Writing – original draft. **Lauren Koscal:** Writing – original draft. **Emma Sears:** Writing – original draft. **Jessica Muniz:** Writing – original draft. **Evelyn Jacovino:** Writing – original draft. **Lauren Thayer:** Writing – original draft. **Todd Allen Lane:** Writing – original draft. **Elizabeth Dos Santos:** Resources.

## Declaration of competing interest

The authors of this manuscript have no competing interest and no conflict of interests to report. Thank you.
